# Somnambulism induced by Hydroxyzine

**DOI:** 10.1192/j.eurpsy.2022.2099

**Published:** 2022-09-01

**Authors:** R. Jomli, H. Jemli, H. Ghabi, S. Madouri, A. Ouertani, U. Ouali

**Affiliations:** 1 Razi Hospital, Psychiatry A, manouba, Tunisia; 2 university of tunis elmanar, Faculty Of Medicine Of Tunis, manouba, Tunisia

**Keywords:** sleep disorder, bipolar disorder, hydroxyzine

## Abstract

**Introduction:**

Somnambulism or sleepwalking could be explained by dysfunction in the regulation of slow-wave sleep. It may be caused by drugs; in the literature, cases of somnambulism that occurred by olanzapine and lithium have been reported.

**Objectives:**

Discuss the association between somnambulism and Hydroxyzine.

**Methods:**

We will discuss the case of a patient with bipolar disorder treated with olanzapine and lithium who experienced episodes of somnambulism after adding Hydroxyzine.

**Results:**

A 42-year-old woman, with no history of somnambulism, followed in our department for a bipolar disorder type 1, treated with 750 mg of lithium and 20 mg of olanzapine. During her usual control, she reported insomnia Hydroxyzine was added at the dose of 50 mg. At the next medical appointment, she said that her husband had noticed that she waked up at night and she eats, she ambulates and searches things. Episodes that the patient did not remember. She was tranferd to the neurolgic departement. She did a neurological exam, an electroencephalogram, and a brain scan, witch were normal. The polysomnography confirmed the diagnosis. The neurologist retained the diagnosis of somnambulism induced by Hdroxizine regarding the chronology of the symptoms. The somnambulism ceased after stopping Hydroxyzine.

**Conclusions:**

Lithium and olanzapine were associated with the occur of somnambulism, but hydroxyzine had never been reported as a somnambulism drug inducing. Drug interaction may explain this phenomenon.

**Disclosure:**

No significant relationships.

